# Whole-Genome Sequences of Five *Burkholderia pseudomallei* Isolates from Australian Cystic Fibrosis Patients

**DOI:** 10.1128/genomeA.00254-15

**Published:** 2015-04-16

**Authors:** Linda T. Viberg, Erin P. Price, Timothy J. Kidd, Scott C. Bell, Bart J. Currie, Derek S. Sarovich

**Affiliations:** a Global and Tropical Health Division, Menzies School of Health Research, Darwin, Northern Territory, Australia; b Queensland Children’s Medical Research Institute, The University of Queensland, Herston, Queensland, Australia; c Center for Infection and Immunity, Queen’s University Belfast, Northern Ireland; d Department of Thoracic Medicine, The Prince Charles Hospital, Chermside, Queensland, Australia; e Department of Infectious Diseases and Northern Territory Medical Program, Royal Darwin Hospital, Darwin, Northern Territory, Australia

## Abstract

We report here five improved high-quality draft genomes of *Burkholderia pseudomallei* isolated from Australian cystic fibrosis (CF) patients. This pathogen is rarely seen in CF patients. These genomes will be used to better understand chronic carriage of *B. pseudomallei* in the CF lung and the within-host evolution of longitudinal isolates from these patients.

## GENOME ANNOUNCEMENT

*Burkholderia pseudomallei* is the causative agent of melioidosis, a potentially lethal disease with multiple clinical presentations, of which pneumonia is most common (1–3). Infection with *B. pseudomallei* is acquired following accidental percutaneous inoculation with contaminated soil or water, or by inhalation or ingestion. Most reported cases of melioidosis are from the regions of northern Australia and Southeast Asia that are highly endemic for *B. pseudomallei*, with sporadic reports from other endemic regions, including the Middle East, Africa, South and Central America, and the Caribbean (4, 5).

Cystic fibrosis (CF) patients typically develop infections caused by a range of opportunistic pathogens, such as *Pseudomonas aeruginosa*, *Staphylococcus aureus*, and *Haemophilus influenzae*, which may be eradicated in the early phases, but with increasing age, these can develop into a chronic infection (6). The factors that promote the development of infection are complex but include the abnormal composition of the airway lining fluid as a consequence of abnormal expression of the CF transmembrane regulator protein (CFTR). *B. pseudomallei* has been identified in the CF lung in a small proportion of CF patients living or traveling to regions endemic for the pathogen (7–12).

*B. pseudomallei* is known to cause chronic infections that can be difficult to treat and, in certain instances, persist for years (13, 14). Although many conventional CF-associated pathogens have been studied in detail, little is known about how *B. pseudomallei* behaves within the CF lung. Reports have demonstrated that, like most *B. pseudomallei* infections, clinical symptoms can present as either acute or chronic disease (9). Here, we present improved high-quality draft genome sequences (15) of the initial *B. pseudomallei* strains isolated from five Australian CF patients with chronic *B. pseudomallei* infection.

DNA was extracted from purified culture, as previously reported (16). The DNA samples were subjected to whole-genome sequencing (WGS) from a paired-end Nextera library and with a ~300-bp insertion size using the Illumina HiSeq 2000 platform (Illumina, Inc., San Diego, CA) at Macrogen, Inc. (Geumcheon-gu, Seoul, Republic of Korea). The genomes of the isolates (excluding MSHR8441) were also sequenced using the 454 Genome Sequencer FLX+ instrument (454 Life Sciences, Branford, CT, USA). The sequence reads were quality-filtered and subsequently assembled via hybrid assembly using MIRA (17), followed by PAGIT (18), SSPACE version 2.0 (19), and GapFiller version 1.10 (20) polishing. MSHR8441 was assembled using Velvet version 1.2.10 (21) instead of MIRA but included the same quality improvement steps listed above. The contigs were reordered against *B. pseudomallei* MSHR1153 (GenBank accession numbers CP009271 and CP009272 [22]) or K96243 (GenBank accession numbers NC_006350 and NC_006351 [23]) using Mauve (24). The contig joins were manually checked for synteny among reference genomes using BLAST and were stitched, if possible. A summary of the final genome assembly statistics is provided in Table 1.

**TABLE 1:** Statistics for the 5 *Burkholderia pseudomallei* draft genome sequences

Strain	Alternate ID	Accession no.	Genome size (bp)	No. of contigs	*N*_50_ (bp)	G+C content (%)
QCMRI_BP07	MSHR5651	JYBG00000000	7,767,989	139	227,078	67.6
QCMRI_BP13	MSHR8436	JYBH00000000	7,356,204	98	241,256	68.0
QCMRI_BP18	MSHR5662	JYBI00000000	7,391,892	83	755,997	68.0
QCMRI_BP28	MSHR8438	JYBJ00000000	7,565,815	161	398,485	67.7
QCMRI_BP32	MSHR8441	JYBK00000000	7,108,439	39	371,315	68.2

These genomes will provide useful reference strains for use in analyses of longitudinal isolates from the same patient and provide novel insights into the within-host evolution and adaptation of *B. pseudomallei* in the CF lung. More broadly, these genomes will be useful for identifying parallel evolutionary mechanisms with other Gram-negative pathogens affecting the CF lung.

### Nucleotide sequence accession numbers. 

The genome accession numbers for the assemblies deposited in DDBJ/ENA/GenBank are listed in Table 1.
